# Supplementary figures and images for: Using in vivo calcium imaging to examine joint neuron spontaneous activity and home cage analysis to monitor activity changes in mouse models of arthritis
Source: Arthritis Res Ther. 2025 Mar 27;27:67. doi: 10.1186/s13075-025-03515-w (PMC11948904; doi:10.1186/s13075-025-03515-w)

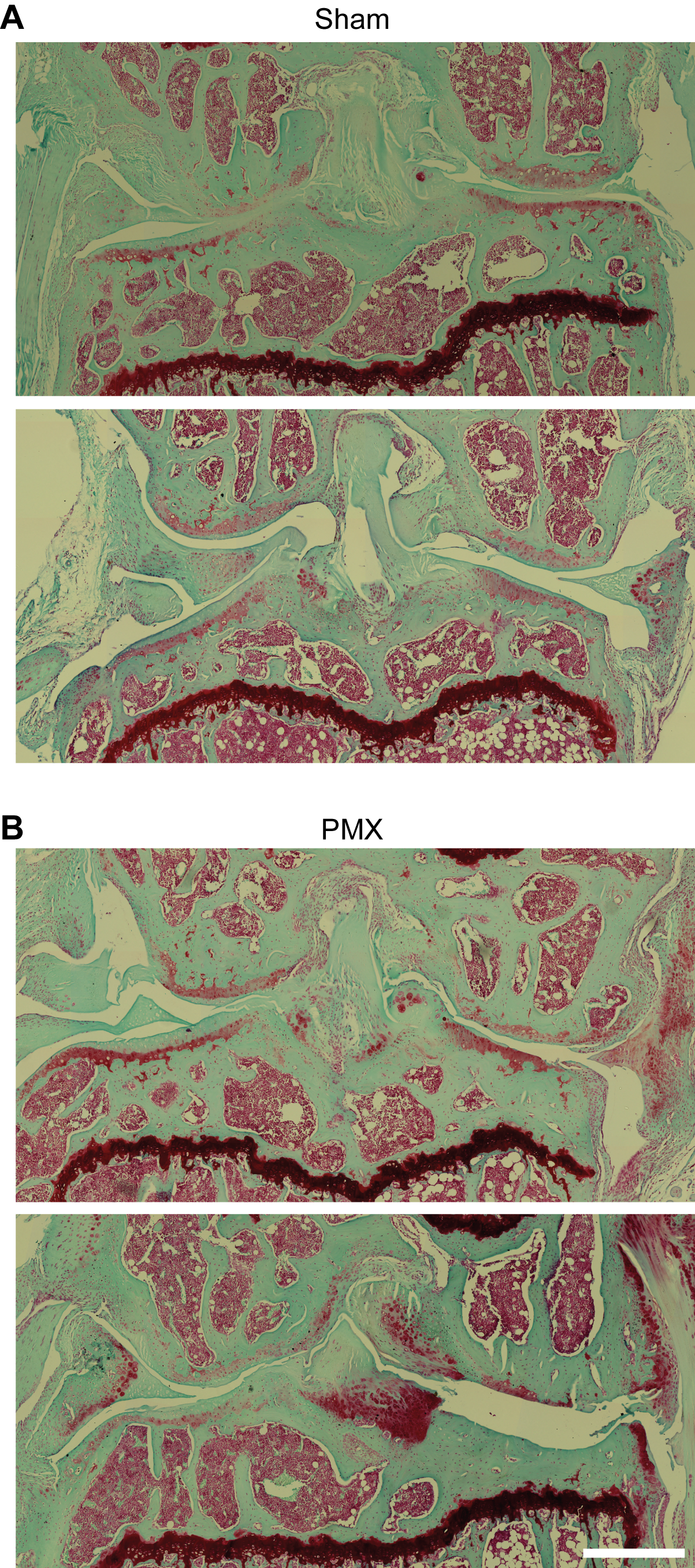

Supplement: Supplementary file 2 — Supplementary Material 2. Supplementary Figure 1. Additional examples of joint histopathology in sham and PMX mice. Example images of Safranin O & Fast Green-stained knee joint sections in Sham (A) and PMX (B) mice at 13-15 weeks post-surgery. Scale bar = 500µm. [file 13075_2025_3515_MOESM2_ESM.tif]

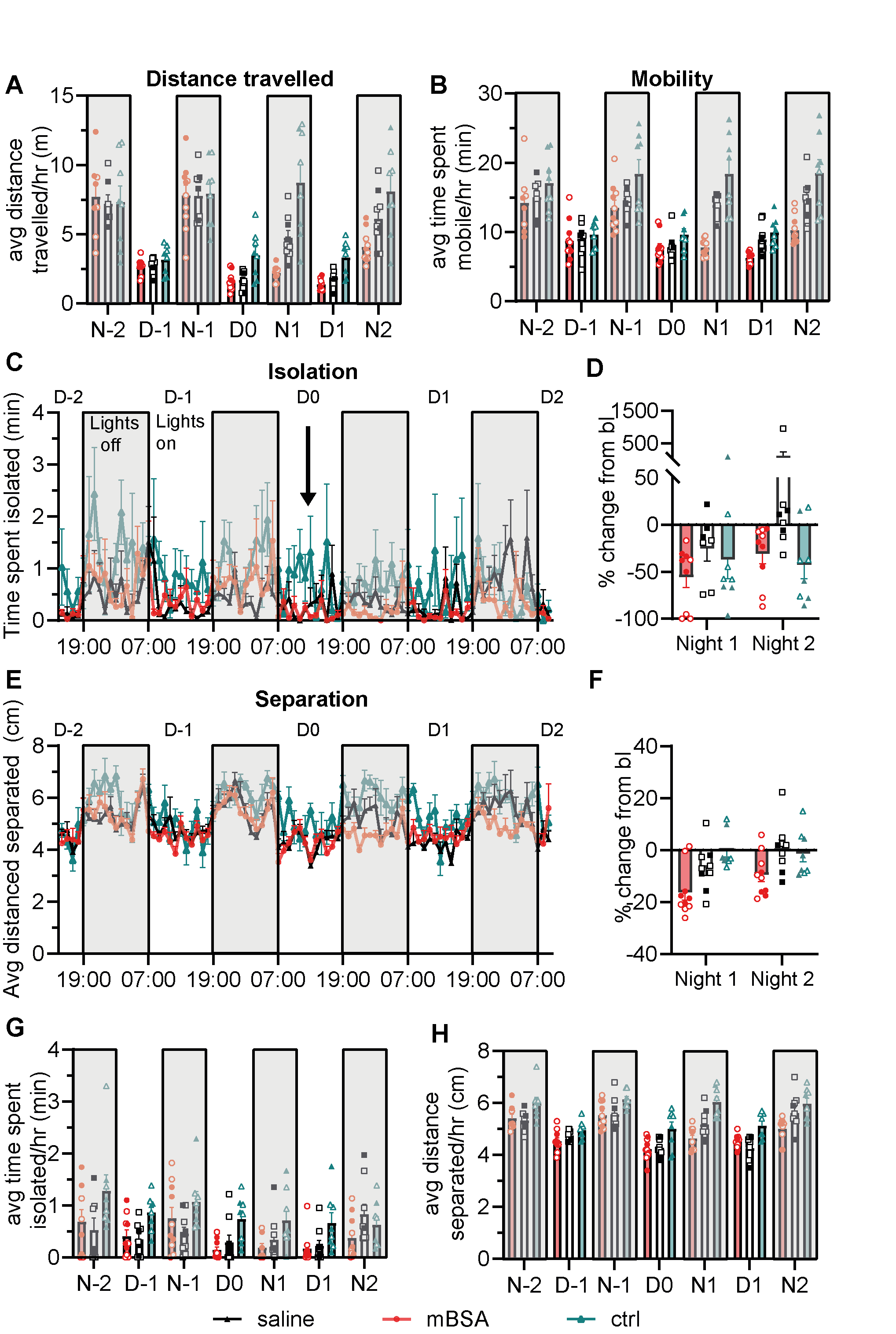

Supplement: Supplementary file 3 — Supplementary Material 3. Supplementary Figure 2. Home cage analyzer data in antigen induced arthritis mice. A&B) Graphs showing distance (A) and mobility (B) data presented in 12-hr time bins. C-E) Graphs showing the time spent isolated (C&D) and the average distance separated (E&F) before and after intraarticular injections (indicated by arrow in C). Data in C) and E) are presented as mean +/- SEM in 1hr intervals. Grey bars represent the dark phase. Data in D) and F) show the percentage change from baseline for the dark phase (19:00-07:00). G&H) Graphs showing isolation (G) and separation (H) data presented in 12-hr time bins. mBSA: n = 10, saline: n = 9. Males = closed circles, females = open circles. [file 13075_2025_3515_MOESM3_ESM.tif]

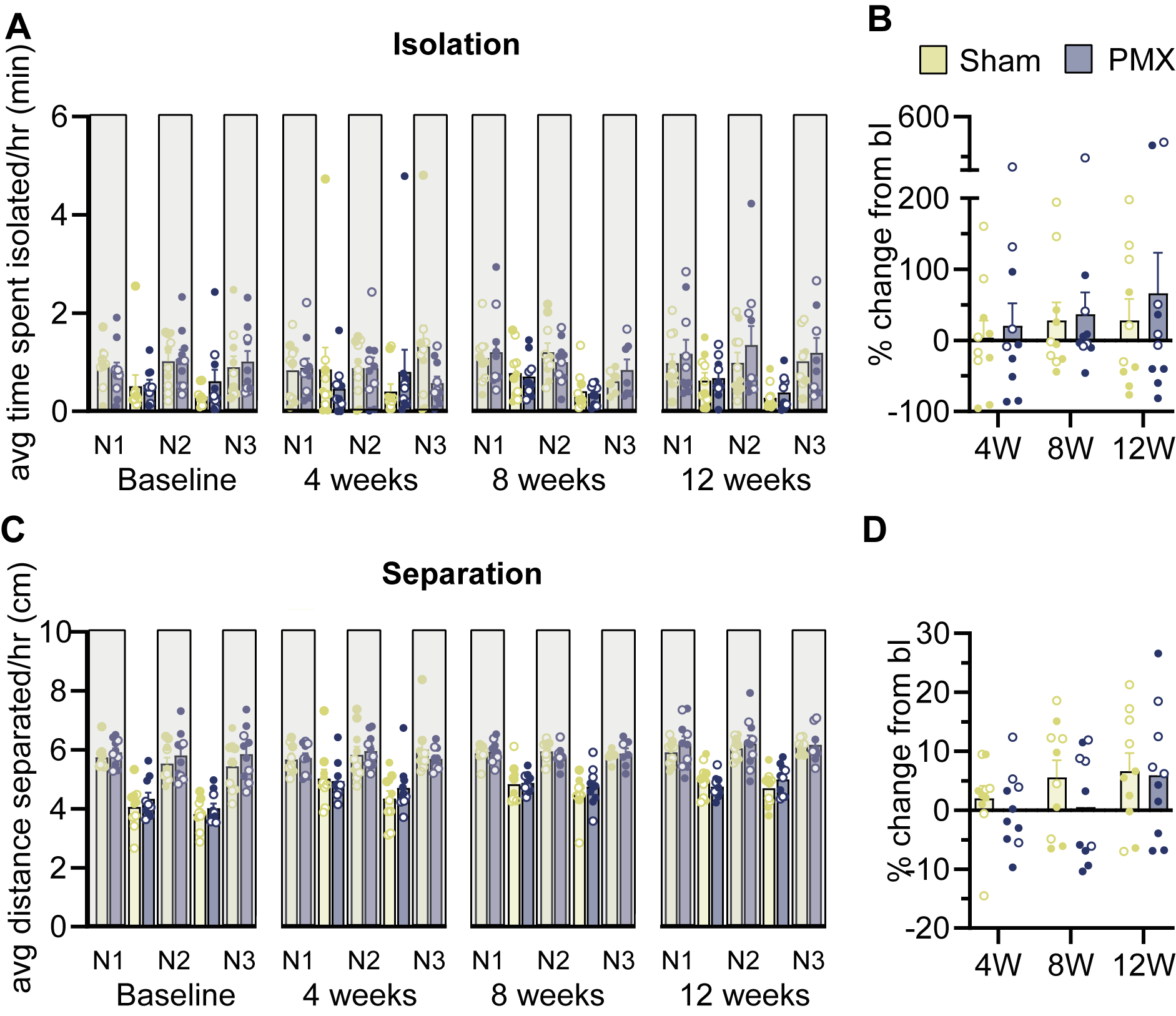

Supplement: Supplementary file 4 — Supplementary Material 4. Supplementary Figure 3. Home cage analyzer data in osteoarthritis mice. A-D) Graphs showing the time spent isolated (A&B) and the average distance separated (C&D) at baseline and at various time intervals post-PMX surgery. Data in A) and C) are presented as mean +/- SEM in 12hr intervals. Grey bars represent the dark phase. Data in B) and D) show the percentage change from baseline for the dark phase (19:00-07:00). PMX: n = 10, sham: n = 10. Males = closed circles, females = open circles. [file 13075_2025_3515_MOESM4_ESM.tif]

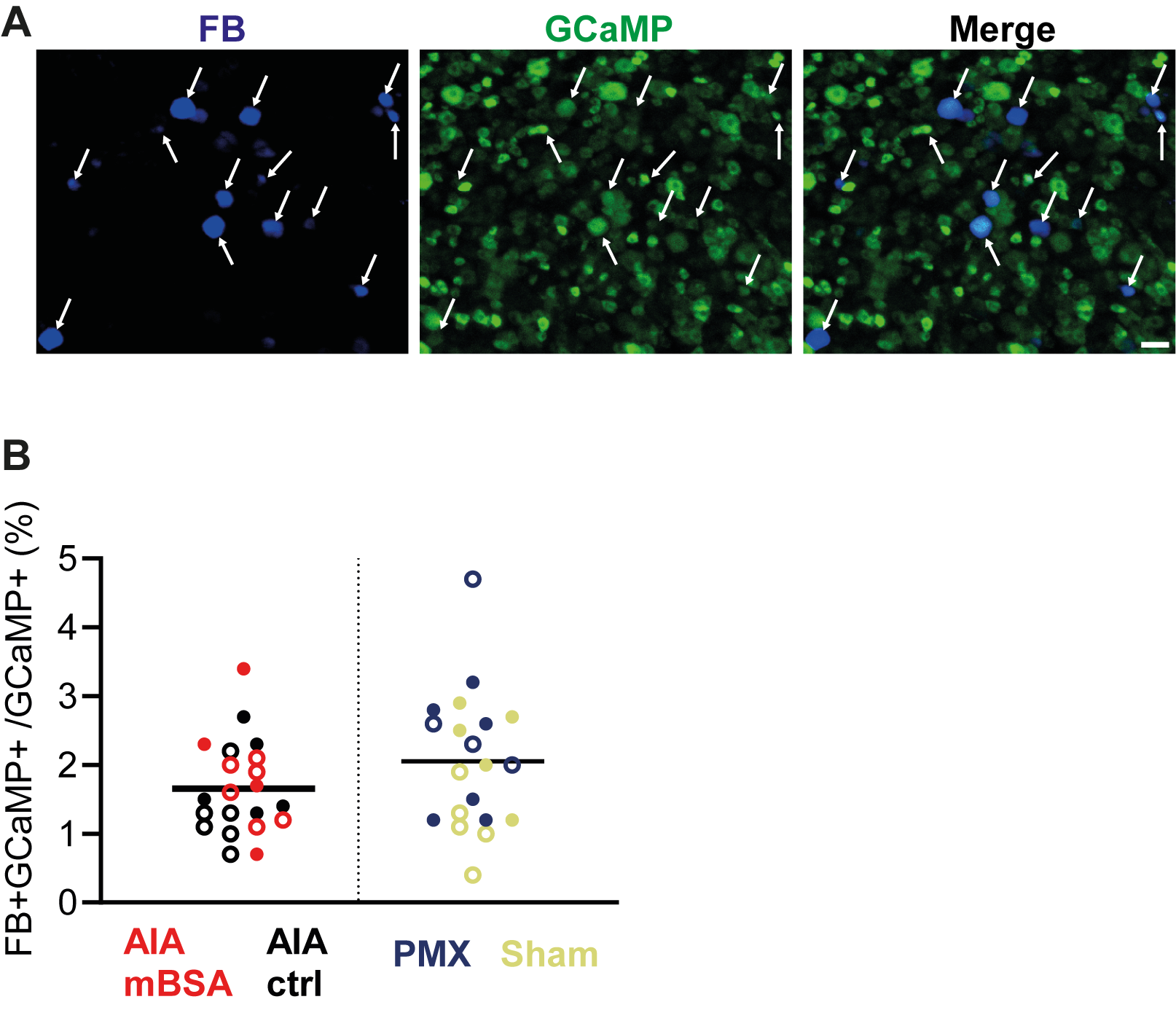

Supplement: Supplementary file 5 — Supplementary Material 5. Supplementary Figure 4. The proportion of GCaMP6s+ neurons labelled with fast blue was similar in AIA and PMX mice. A) Example image showing max projection from a z-stack taken in blue (fast blue) and green (GCaMP6s) channels on the confocal microscope. Arrows indicate neurons that are labelled with fast blue (FB) and GCAMP6s. Scale bar = 50µm. B) Graph showing the proportion of GCaMP+ neurons labelled with fast blue for each experimental group. males = closed circles, females = open circles. [file 13075_2025_3515_MOESM5_ESM.tif]

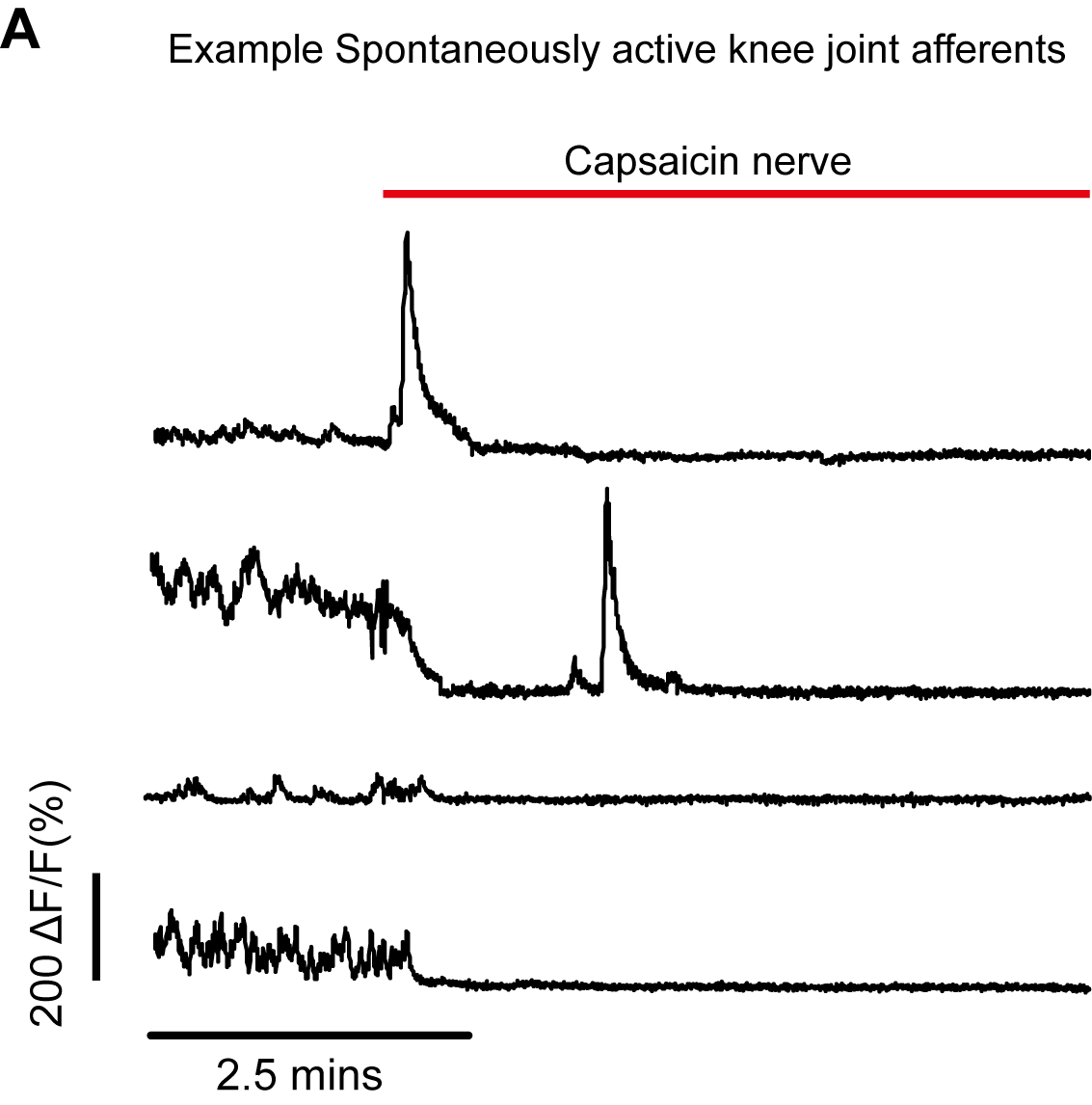

Supplement: Supplementary file 6 — Supplementary Material 6. Supplementary Figure 5. Capsaicin application to the nerve produced variable responses. A) Example traces from spontaneously active joint neurons before and after application to the nerve. Note that activity in some neurons was blocked following capsaicin-induced depolarization (upper trace) whereas others were blocked without any prior depolarization (lower two traces). [file 13075_2025_3515_MOESM6_ESM.tif]

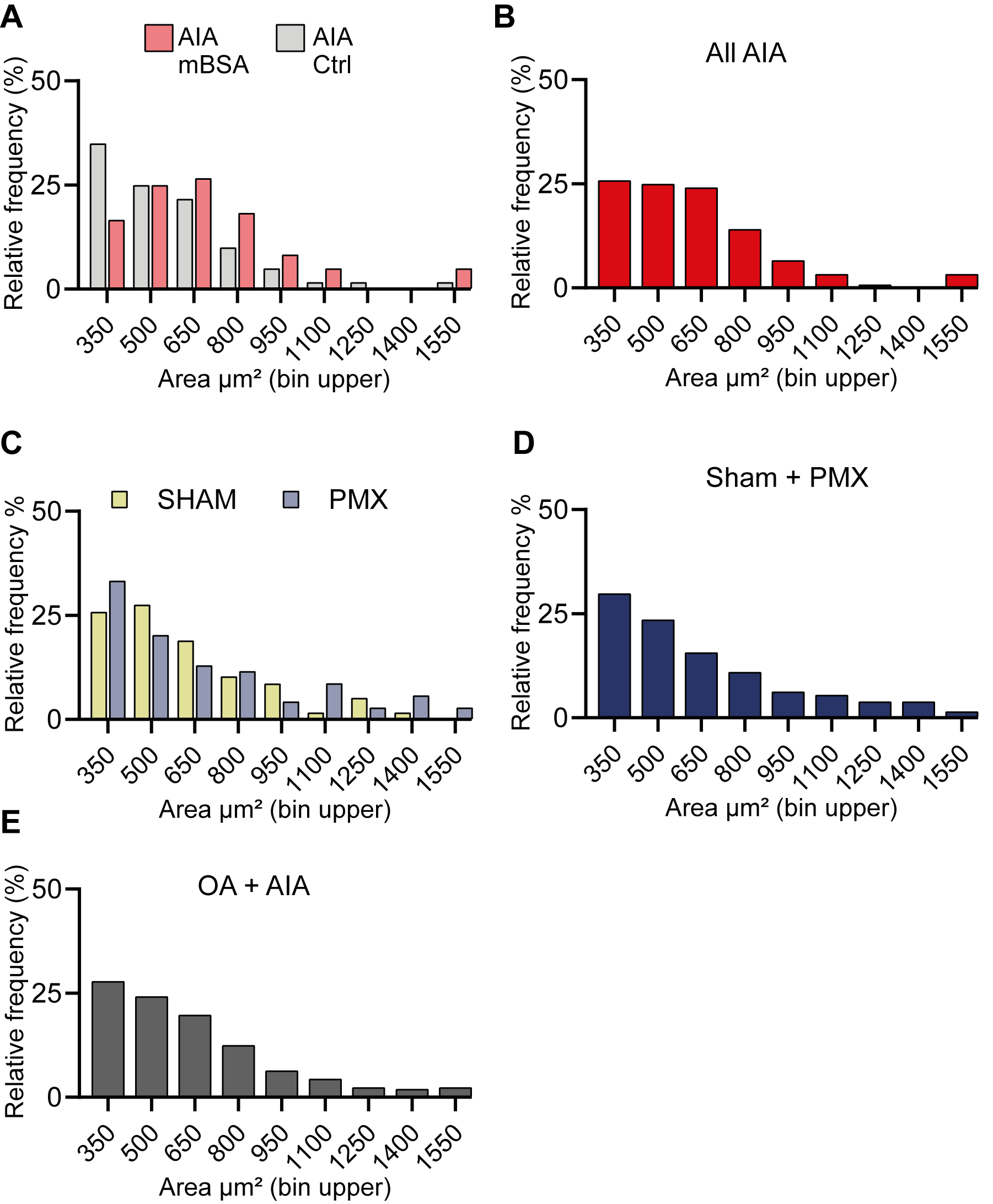

Supplement: Supplementary file 7 — Supplementary Material 7. Supplementary Figure 6. Size histograms for antigen induced arthritis and partial medial meniscectomy mouse cohorts. A-E) Size histogram showing the relative frequency of fast blue (FB+) joint neurons in bin widths of 150 µm². AIA cohorts: AIA mBSA = 63 neurons from n = 10 mice. AIA Ctrl = 61 neurons from n = 11 mice. All AIA = 124 neurons from n = 21 mice. PMX cohorts: Sham = 58 neurons from n = 10 mice. PMX = 71 neurons from n = 10 mice. SHAM+PMX = 129 neurons from n = 20 mice. [file 13075_2025_3515_MOESM7_ESM.tif]
