# Supplementary material for: Using in vivo calcium imaging to examine joint neuron spontaneous activity and home cage analysis to monitor activity changes in mouse models of arthritis
Source: Arthritis Res Ther. 2025 Mar 27;27:67. doi: 10.1186/s13075-025-03515-w (PMC11948904; doi:10.1186/s13075-025-03515-w)
Supplement: Supplementary file 8 — Supplementary Material 8. Supplementary tables 1-3 are contained within the Supplementary tables.doc. [file 13075_2025_3515_MOESM8_ESM.docx]

**Supplementary Table 1.**

|  | Primary outcome measure | Statistical test | # Groups | Animals/ group | Observed effect size | Sensitivity analysis for row sample size/test |  |
| --- | --- | --- | --- | --- | --- | --- | --- |
| AIA knee swelling | Size across knee joint | Independent samples t-test | 2 | 11 | d=2.5* | d=1.26 | |
| PMX histology | Cartilage loss | RM-ANOVA, between-group effect of treatment | 2 | 9-10 | F=1.04* | F=0.56 | |
| AIA  imaging | % joint silent nociceptors spontaneously active | Kruskal-Wallis test | 2 | 10-11 | F=0.72* | F=0.66 | |
| PMX  imaging | % joint silent nociceptors spontaneously active | Kruskal-Wallis test | 2 | 9-10 | F=0.08 | F=0.66 | |
| AIA  behaviour | Distance travelled  Time spent mobile | RM-ANOVA, between-group effect of treatment | 2 | 9-10 | F=1.27* | F=0.55 | |
| PMX  home cage behavior | Distance travelled  Time spent mobile | RM-ANOVA, between-group effect of treatment | 2 | 10 | F=0.37 | F=0.52 | |
| PMX  Weight bearing | Hind limb weight bearing ratio | RM-ANOVA, between-group effect of treatment | 2 | 10 | F=0.39 | F=0.50 | |

Summary of statistical tests and n-numbers used and the effect sizes we observed. A star indicates when an effect size was statistically significant in a given experiment. The last column provides a sensitivity analysis, indicating the minimum effect sizes that can theoretically be detected with 80% probability when carrying out an experiment with the statistical test and n number provided for each row.

**Supplementary Table 2. *In vivo* calcium imaging data presented per neuron.**

|  | AIA | CTRL | PMX | Sham |
| --- | --- | --- | --- | --- |
| # L4 neurons | 3483 | 4082 | 3197 | 3124 |
| # Spontaneously active L4 neurons | 414 | 379 | 227 | 284 |
| # FB+ neurons | 63 | 61 | 75 | 55 |
| # Spontaneously active FB+ neurons | 14 | 1 | 2 | 2 |
| Proportion of spontaneously active L4 neurons still active post-lidocaine | 62/3179 | 64/2522 | 51/3197 | 36/2430 |
| Proportion of spontaneously active FB+ neurons still active post-lidocaine | 1/11 | 0/1 | 0/2 | 0/2 |

FB = fast blue. AIA = Antigen induced arthritis. PMX = partial medial meniscectomy

**Supplementary Table 3. Responses of spontaneously active fast blue joint neurons to capsaicin**

|  | Spontaneously active prior to capsaicin application | Blocked after capsaicin | Activated and blocked after capsaicin | Activated by capsaicin |
| --- | --- | --- | --- | --- |
| # neurons | 8 | 6 | 2 | 2 |
| % |  | 75.0% | 25.0% | 25.0% |

Data from n=5 mice that had capsaicin applied to the nerve.
